# *Staphylococcus aureus* autoinducer-2 quorum sensing decreases biofilm formation in an *icaR*-dependent manner

**DOI:** 10.1186/1471-2180-12-288

**Published:** 2012-12-05

**Authors:** Dan Yu, Liping Zhao, Ting Xue, Baolin Sun

**Affiliations:** 1 Department of Microbiology and Immunology, School of Life Sciences, University of Science and Technology of China, Hefei, Anhui, 230027, China

## Abstract

**Background:**

*Staphylococcus aureus* is an important pathogen that causes biofilm-associated infection in humans. Autoinducer 2 (AI-2), a quorum-sensing (QS) signal for interspecies communication, has a wide range of regulatory functions in both Gram-positive and Gram-negative bacteria, but its exact role in biofilm formation in *S. aureus* remains unclear.

**Results:**

Here we demonstrate that mutation of the AI-2 synthase gene *luxS* in *S. aureus* RN6390B results in increased biofilm formation compared with the wild-type (WT) strain under static, flowing and anaerobic conditions and in a mouse model. Addition of the chemically synthesized AI-2 precursor in the *luxS* mutation strain (ΔluxS) restored the WT phenotype. Real-time RT-PCR analysis showed that AI-2 activated the transcription of *icaR*, a repressor of the *ica* operon, and subsequently a decreased level of *icaA* transcription, which was presumably the main reason why *luxS* mutation influences biofilm formation. Furthermore, we compared the roles of the *agr*-mediated QS system and the LuxS/AI-2 QS system in the regulation of biofilm formation using the ΔluxS strain, RN6911 and the Δagr ΔluxS strain. Our data indicate a cumulative effect of the two QS systems on the regulation of biofilm formation in *S. aureus*.

**Conclusion:**

These findings demonstrate that AI-2 can decrease biofilm formation in *S. aureus* via an *icaR*-activation pathway. This study may provide clues for therapy in *S. aureus* biofilm-associated infection.

## Background

*Staphylococcus aureus* is an opportunistic pathogen that can adhere to many tissues and implants in humans to form biofilms causing refractory chronic infections [1,2]. Many therapies have been proposed but the potential efficacy is limited [3]. Given this situation, intensive research into the molecular mechanism of biofilm formation in *S. aureus* could facilitate the development of novel therapeutic devices.

Biofilms are complex communities of microorganisms encased in slime that can attach to surfaces [4]. Protein, polysaccharide, and extracellular DNA are supposed to be important components of *Staphylococcal* biofilms [5-7]. Biofilm formation is established using at least two properties: the adherence of cells to a surface and accumulation to form multi-layered cell clusters [8,9]. The latter process is closely related to polysaccharide intercellular adhesion (PIA), a polysaccharide composed of β-1,6-linked *N*-acetylglucosamine residues in *Staphylococci*[10]. The intercellular adhesion (*ica*) locus is composed of four open reading frames (ORFs) *icaA*, *icaD*, *icaB* and *icaC* in an operon [11,12], and is responsible for generating PIA, which is required for biofilm formation in *S. aureus*. Moreover, decreased PIA level is considered to be the main factor leading to the destructive ability of biofilm formation in *S. aureus* RN6390B [13]. In recent years, many factors including glucose, glucosamine, oleic acid, urea, anaerobiosis and iron limitation have been identified as influencing the expression of PIA [12,14-18]. In addition, it has been demonstrated that IcaR represses *ica* expression by binding to the *icaA* promoter region [19]. Furthermore, QS has been recently shown to control the expression of the *ica* operon [20].

Quorum sensing is a widespread system used by bacteria for cell-to-cell communication, which regulates expression of multiple genes in a cell density-dependent manner [21,22]. The unique QS system shared by Gram-positive and Gram-negative bacteria is mediated by AI-2 [23], which is a signalling molecule synthesized by the *luxS* gene [24,25]. AI-2 originates from the auto-cyclization of precursor 4, 5-dihydroxy-2, 3-pentanedione (DPD) [26,27], and has been reported to regulate luminescence, motility and virulence [28-30]. Biofilm formation is known as the "bacterial social behaviour", in part owing to an organised mode of growth in a hostile environment. Many studies have described the role of AI-2 in biofilm formation. For example, synthetic AI-2 directly stimulates *Escherichia coli* biofilm formation and controls biofilm architecture by stimulating bacterial motility [31]. Subsequently, several studies also indicated that AI-2 indeed controls biofilm formation [32-34]. In contrast, some researchers reported that addition of AI-2 failed to restore biofilm phenotype of the parental strain [35-40], owing to the central metabolic effect of LuxS or difficulty in complementation of AI-2 [41]. There exists a conserved *luxS* gene in *S. aureus*, and it has been proved to be functional for generating AI-2 [42]*.* Previous work indicated that AI-2-mediated QS modulated capsular polysaccharide synthesis and virulence in *S. aureus*[43], deletion of the *luxS* gene led to increased biofilm formation in *Staphylococcus epidermis*[20], and biofilm enhancement due to *luxS* repression was manifested by an increase in PIA [44].

In this study, we provide evidence that *S. aureus* ΔluxS strain formed stronger biofilms than the WT strain RN6390B, and that the *luxS* mutation was complemented by adding chemically synthesized DPD, the exogenous precursor of AI-2. AI-2 activated the transcription of *icaR*, and subsequently led to decreased *icaA* transcription, as determined by real-time RT-PCR analysis. Furthermore, the differences in biofilm-forming ability of *S. aureus* RN6911, ΔluxS strain, and the ΔagrΔluxS strain were also investigated. Our data suggest that AI-2 could inhibit biofilm formation in *S. aureus* RN6390B through the IcaR-dependent regulation of the *ica* operon.

## Methods

### Bacterial strains, plasmids and DNA manipulations

The bacterial strains and plasmids used in this study are described in Table 1. *E. coli* cells were grown in Luria-Bertani (LB) medium (Oxoid) with appropriate antibiotics for cloning selection. *S. aureus* strain RN4220, a cloning intermediate, was used for propagation of plasmids prior to transformation into other *S. aureus* strains. *S. aureus* cells were grown at 37°C in tryptic soy broth containing 0.25% dextrose (TSBg) (Difco No. 211825). In the flow cell assay, biofilm bacteria were grown in tryptic soy broth without dextrose (TSB) (Difco No. 286220). Medium was supplemented when appropriate with ampicillin (150 μg/ml), kanamycin (50 μg/ml), erythromycin (2.5 μg/ml) and chloramphenicol (15 μg/ml).

**Table 1:** Strains and plasmids used in this study

**Strain or plasmid**	**Description**	**Reference or source**
RN6390B	Standard laboratory strain	NARSA^a^
RN4220	8325-4 r^-^	NARSA
ΔluxS	RN6390B *luxS*::*ermB*	This study
RN6911	RN6390B derivative; agr locus replaced with tetM cassette	NARSA
ΔagrΔluxS	RN6911 *luxS*::*ermB, agr/luxS* double mutant	This study
ΔluxSpluxS	Complemented strain of ΔluxS; Ap^r^ Cm^r^	This study
RN6390BG	RN6390B/pgfp	This study
ΔluxSG	ΔluxS/pgfp	This study
RN6911G	RN6911/pgfp	This study
ΔagrΔluxSG	ΔagrΔluxS/pgfp	This study
NCTC8325	Standard Laboratory strain	NARSA
NCTC8325ΔluxS	NCTC8325 *luxS*::*ermB*	60
*E. coli* strains		
TOP10	Cloning	Invitrogen
Plasmids		
pEASY-Blunt	Clone vector, Kan^r^ Ap^r^	Transgen
pBTluxS	Vector used for *luxS* mutagenesis, Ap^r^ Cm^r^ Em^r^	60
pLI50	*E. coli*-*S. aureus* shuttle cloning vector, Ap^r^ Cm^r^	Addgene
pLIluxS	pLI50 with *luxS* and its promoter, Ap^r^ Cm^r^	60
pgfp	*gfp* expression with the promoter of S10 ribosomal gene, Ap^r^, Cm^r^	

^a^*NARSA*, Network on Antimicrobial Resistance in *Staphylococcus aureus*.

### Construction of bacterial strains

To construct the ΔluxS strain from *S. aureus* RN6390B and the Δagr ΔluxS strain from *S. aureus* RN6911, the purified pBTluxS plasmid was used for allele replacement by erythromycin-resistance gene insertional mutagenesis as described previously [45]. Briefly, the appropriate upstream and downstream fragments of *luxS* were amplified from the genome of RN6390B, and the erythromycin-resistance gene was amplified from pEC1 with the relevant primers. The three fragments were ligated with each other with the erythromycin-resistance gene in the middle, and then ligated with the temperature-sensitive shuttle vector pBT2. The resulting plasmid pBTluxS [43] was introduced by electroporation into *S. aureus* strain RN4220 for propagation, and then transformed into *S. aureus* RN6390B for *luxS* mutation and *S. aureus* RN6911 for *agr luxS* double-gene mutation. All primers used in this study are listed in Table 2.

**Table 2:** Oligonucleotide primers used in this study

**Primer**	**Sequence**
rt-16S-f	CGTGGAGGGTCATTGGA
rt-16S-r	CGTTTACGGCGTGGACTA
rt-icaA-f	TTTCGGGTGTCTTCACTCTAT
rt-icaA-r	CGTAGTAATACTTCGTGTCCC
rt-icaR-f	ATCTAATACGCCTGAGGA
rt-icaR-r	TTCTTCCACTGCTCCAA
rt-clfB-f	TTTGGGATAGGCAATCATCA
rt-clfB-r	TCATTTGTTGAAGCTGGCTC
rt-fnbA-f	ATGATCGTTGTTGGGATG
rt-fnbA-r	GCAGTTTGTGGTGCTTGT
rt-fnbB-f	ACAAGTAATGGTGGGTAC
rt-fnbB-r	AATAAGGATAGTATGGGT
rt-map-f	AAACTACCGGCAACTCAA
rt-map-r	TGTTACACCGCGTTCATC
rt-efb-f	TAACATTAGCGGCAATAG
rt-efb-r	CCATATTCGAATGTACCA

To make the *luxS*-complemented strain, the pLIluxS plasmid, which contains the native promoter of *luxS* and its intact open reading frame, was constructed in our previous work [43]. We purified the pLIluxS plasmid and transformed it into the ΔluxS strain for complementation, thus constructing the ΔluxSpluxS strain. WT and ΔluxS strains were also transformed with the empty plasmid pLI50 constructing strains WTp and ΔluxSp, which were used as the control. These strains transformed with plasmid were cultured in medium with chloramphenicol (15 μg/ml). The AI-2 precursor molecule, DPD, of which the storage concentration is 3.9 mM dissolved in water, was purchased from Omm Scientific Inc., TX, USA. 

### Biofilm formation and analysis

Biofilm formation under static conditions was determined by the microtitre plate assay based on the method described previously [46]. Briefly, the overnight cultures were made at a 1:100 dilution using fresh TSBg. The diluted cell suspension was inoculated into flat-bottom 24-well polystyrene plates (Costar 3599, Corning Inc., Corning, NY), 1 ml for each well. The plates were incubated at 37°C for different time courses and the wells were rinsed gently with water five times to remove non-adherent cells. Subsequently, the plates were stained with 0.5% crystal violet for 15 m, and then rinsed again with water to remove unbound stain. After that, the plates were dried, and the optical density at 560 nm (OD_560_) was determined with an enzyme-linked immunosorbent assay reader in a 5 × 5 scan model. To investigate the effect of AI-2, the medium was supplemented with chemically synthesized DPD with a concentration range of 0.39 nM to 390 nM.

Biofilm formation was also examined in a flow cell (Stovall, Greensboro, USA), which was assembled and prepared according to the manufacturer's instructions. Flow cell experiments and laser scanning confocal microscope (CLSM) were performed as described previously [47]. Overnight cultures of different strains were adjusted to OD_600_ of 6.5 and made at a 1:100 dilution in fresh 2% TSB. Flow cells were inoculated with 4 ml of these culture dilutions and incubated at 37°C for 1 h, and then laminar flow (250 μl/m) was initiated. Biofilms of different strains were cultivated at 37°C in 2% TSB in three individual channels. The strains were transformed with the GFP plasmid for fluorescence detection, thus chloramphenicol was added to the flow cell medium to maintain plasmid selection. CLSM was performed on a Zeiss LSM710 system (Carl Zeiss, Jena, Germany) with a 20 × 0.8 n.a. apochromatic objective. Z-stacks were collected at 1 μm intervals. Confocal parameters set for WT biofilm detection were taken as standard settings. Selected confocal images stood for similar areas of interest and each confocal experiment was repeated four times. The confocal images were acquired from Zeiss ZEN 2010 software package (Carl Zeiss, Jena, Germany) and the three-dimensional biofilm images were rendered with Imaris 7.0 (Bitplane, Zurich, Switzerland). Biofilm biomass and average thickness were analysed with the COMSTAT program [48] and were indicated as the mean ± standard deviation calculated from three images obtained from a given biofilm.

### Ethical statement

The use and care of mice in this study was performed strictly according to the Institutional Animal Care and Use Committee guideline of University of Science and Technology of China (USTCACUC1101053).

### *In vivo* model of catheter-associated biofilm formation

Biofilm formation was assessed *in vivo* using a murine model of catheter-associated infection [49]. Briefly, male BALB/c mice (6- to 8-weeks old) were obtained from Shanghai Laboratory Animal Centre of Chinese Academy of Sciences (Shanghai, China). The mice were anaesthetised with 1% pentobarbital sodium (0.01 ml/g of body weight) and surgically dissected. Specifically, a 1-cm 18G FEP polymer catheter (Introcan, Melsungen, Germany) was implanted subcutaneously in the dorsal area of the mice. The wound was closed with surgical glue. After incubation of 24 h, 5 × 10^7^ colony-forming units (CFU) of the test strains in a total volume of 100 μl were introduced directly into the lumen of the catheters. Mice were euthanised after 3 days of infection, and then the catheters were removed carefully and washed briefly with phosphate-buffered saline (PBS). Catheters were placed in 1 ml of sterile PBS and sonicated for 30 s to remove the adherent bacteria. The number of bacteria was determined by plating on tryptic soy agar (TSA).

### Anaerobic conditions

Biofilm formation was also monitored under anaerobic conditions. The Forma Anaerobic System (Thermo, Waltham, USA) was used to provide strictly anaerobic conditions for bacterial growth and related operations. Overnight cultures were adjusted to OD_600_ of 6.5, and then the bacterial cultures were carried into the anaerobic system for 1:100 dilution and inoculated into 24-well plates. Resazurin, which is used as an indicator for anaerobic conditions, was added to final concentration of 0.0002% (w/v). The plates were incubated at 37°C for 4 h and OD_560_ was determined after crystal violet staining.

A standard anaerobic jar of 120 ml volume was used to monitor the biofilm formation of the WT strain and the mutants under anaerobic conditions. Medium and containers with thorough scavenging were prepared as follows. Water was boiled using a three-necked bottle to degas the water while nitrogen was bubbled into the bottle to keep the contents anaerobic. TSBg medium was prepared with this degassed water. Then each anaerobic jar was dispensed with 50 ml TSBg while nitrogen was gassed into the jar to drive out the oxygen. The rubber plug was quickly stuffed up following by an aluminium cap added, and then the jar containing TSBg was autoclaved at 121°C, 15 m. After preparation of the medium, biofilm formation under anaerobic conditions was examined and the operations were carried out in the anaerobic system.

### Scanning electron microscopy (SEM)

Biofilm bacteria were grown on coverslips for five days, and then the coverslips were cut from the flow-cell settings and immediately fixed with 2.5% (vol/vol) glutaraldehyde in Dulbecco PBS (pH 7.2) overnight. According to the methods described previously [50], the coverslips were rinsed with PBS three times and dehydrated through an ethanol series (30%, 50%, 75%, 85% and 95%). Samples were dried and gold-palladium coated prior to SEM examination and micrographs were made with a XL-30 SEM at × 1500 to × 5000 magnification (FEI, Hillsboro, USA).

### RNA isolation and real-time RT-PCR

All the bacteria used for RNA isolation to investigate the expression of genes that affect biofilm formation were those that grew statically in the 24-well plate. Bacteria in the wells of biofilm formation at different time courses (4 h, 8 h, 12 h) were collected and re-suspended in TE (Tris-EDTA) buffer (pH 8.0) containing 10 g/l lysozyme and 40 mg/l lysostaphin. After incubation at 37°C for 8 m, *S. aureus* cells were prepared for total RNA extraction using the Trizol method (Invitrogen), and the residual DNA was removed with RNase-free DNase I (TaKaRa). The concentration of RNA was adjusted to 100 ng/μl, and the samples were stored at −70°C. cDNA templates were synthesized from 50 ng RNA with PrimeScript™ 1st strand cDNA Synthesis Kit (TaKaRa) and gene-specific primers at 42°C for 15 m, 85°C for 5 s. Real-time PCR was performed with the cDNA and SYBR Premix Ex Taq (TaKaRa) using a StepOne Real-Time PCR System (Applied Biosystems). The quantity of cDNA measured by real-time PCR was normalised to the abundance of 16S cDNA. Real-time RT-PCR was repeated three times in triplicate parallel experiments.

### Statistical analysis

The paired *t* test was used for statistical comparisons between groups. The level of statistical significance was set at a P value of ≤ 0.05.

## Results

### AI-2 inhibits biofilm formation in a concentration-dependent manner under static conditions

**Figure 1:**
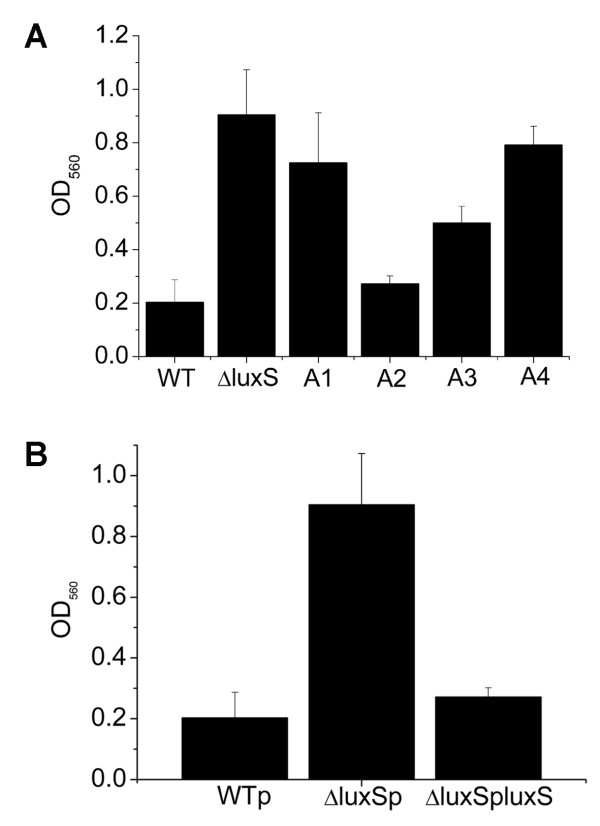
**Biofilm formation under static conditions and chemical complementation by DPD of different concentrations.** Biofilm growth of *S. aureus* WT (RN6390B), ΔluxS and ΔluxS complemented with different concentrations of chemically synthesized DPD in 24-well plates for 4 h under aerobic conditions (A1: 0.39 nM, A2: 3.9 nM, A3: 39 nM, A4: 390 nM). The cells that adhered to the plate after staining with crystal violet were measured by OD_560_ .

Previous studies showed that biofilm formation was influenced by the LuxS/AI-2 system both in Gram-positive and Gram-negative bacteria [32,34]. The genome of *S. aureus* encodes a typical *luxS* gene, which plays a role in the regulation of capsular polysaccharide synthesis and virulence [43]. In this study, to investigate whether LuxS/AI-2 system regulates biofilm formation in *S. aureus*, we monitored the biofilm formation of *S. aureus* WT strain RN6390B and the isogenic derivative ΔluxS strain using a microtitre plate assay. As shown in Figure 1A, the WT strain formed almost no biofilm after 4 h incubation at 37°C. However, the ΔluxS strain formed strong biofilms as measured by quantitative spectrophotometric analysis based on OD_560_ after crystal violet staining (Figure 1A). This discrepancy could be complemented by introducing a plasmid that contains the *luxS* gene (Figure 1B).

The effects of LuxS could be attributed to its central metabolic function or the AI-2-mediated QS regulation, which has been reported to influence biofilm formation in some strains [32-34]. To determine if AI-2, as a QS signal, regulates biofilm formation in *S. aureus*, the chemically synthesized pre-AI-2 molecule DPD at concentrations from 0.39 nM to 390 nM was used to complement the ΔluxS strain. The resulting data suggested that exogenous AI-2 could decrease biofilm formation of the ΔluxS strain and the effective concentration for complementation was from 3.9 nM to 39 nM DPD (Figure 1A). As expected, these concentrations were within the range that has been reported [51]. The phenomenon that the higher concentration of AI-2 does not take effect on biofilm formation is very interesting, which has also been found in other species [51]. In the previous work [52,53], they indicated that AI-2 activity was associated with cyclic derivatives of this molecule that can be generated spontaneously. Therefore, it is possible that the concentration of effective molecules is different as the DPD concentration changes. These findings indicate that AI-2 could complement the effect of *luxS* mutation on biofilm formation and act in a concentration-dependent manner in *S. aureus*.

### AI-2 inhibits biofilm formation in flow cell

**Figure 2:**
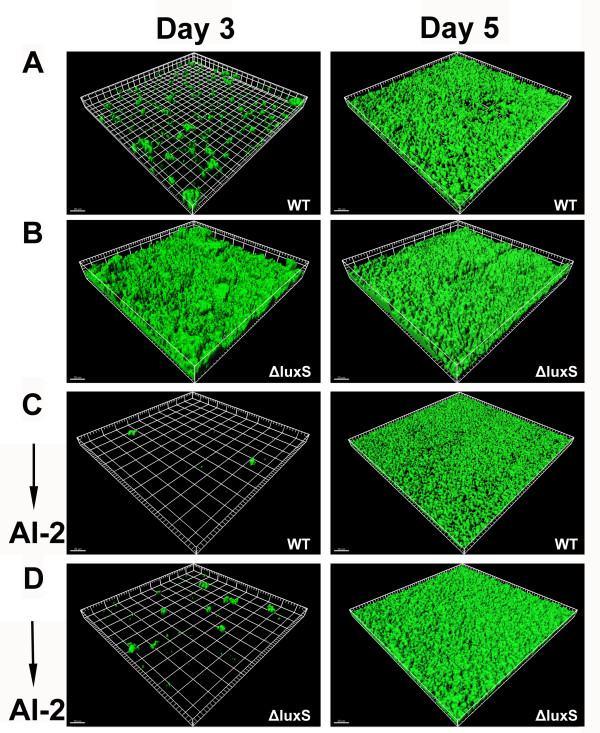
**Biofilm formation in flow cell and chemical complementation by DPD.** Biofilms of WT (RN6390BG) and ΔluxS (ΔluxSG) were grown in a flow cell in 2% TSB with chloramphenicol (15 μg/ml). Biofilm integrity and GFP fluorescence were monitored at the 3rd day and the 5th day by CLSM. For chemical complementation, 3.9 nM DPD was added to the TSB medium at the beginning of the experiment. CLSM images are representative of two separate experiments and each grid square represents 20 μm (**A**) WT. (**B**) ΔluxS. (**C**) WT supplemented with DPD. (**D**) ΔluxS supplemented with DPD.

To further compare the different biofilm formation ability owing to *luxS* deletion, biofilm formation of WT and the ΔluxS strains was assessed using a flow-cell assay. After 3 days of incubation, biofilms produced by WT strain were undetectable as monitored by CLSM. In contrast, the ΔluxS strain began to form intact and rough biofilms. At the 5th day, the WT strain produced biofilms similar to that formed by the ΔluxS strain 2 days before; meanwhile, the ΔluxS strain formed thicker and stronger biofilms (Figure 2A and B). Analysis of the biofilms by COMSTAT is shown in Table 3. The ΔluxS strain exhibited significantly increased total biomass and average thickness of biofilms relative to those of the WT strain.

**Table 3:** Biofilm formation of WT and ΔluxS strains

**Strains**	**Biofilm biomass (μm**^**3**^**/μm**^**2**^**)**		**Average thickness (μm)**	
	**Day 3**	**Day 5**	**Day 3**	**Day 5**
WT	3.01 ± 0.2	11.71 ± 1.25	3.81 ± 0.35	11.51 ± 0.92
ΔluxS	20.16 ± 1.59*	25.67 ± 1.16*	20.79 ± 1.47*	26.18 ± 0.43*
WT + AI-2	0.11 ± 0.01	10.44 ± 0.51	0.12 ± 0.01	9.45 ± 0.5
ΔluxS + AI-2	0.49 ± 0.018	14.31 ± 0.59	0.59 ± 0.06	13.53 ± 0.5

* Significantly different results compared with WT (P < 0.01).

In the flow-cell assay, 3.9 nM DPD was added to the culture medium at the beginning of the experiment. As expected, examination with CLSM showed that the ΔluxS strain complemented with 3.9 nM DPD did not produce biofilms after 3 days of growth in the flow cell, and formed biofilms similar to that of the WT strain at the 5th day (Figure 2C and D). As shown in Table 3, they both formed ~10-μm thick biofilms until the 5th day. These results suggest that AI-2 supplementation decreases biofilm formation under flow conditions.

### Inactivation of *luxS* results in increased biofilm formation *in vivo*

**Figure 3:**
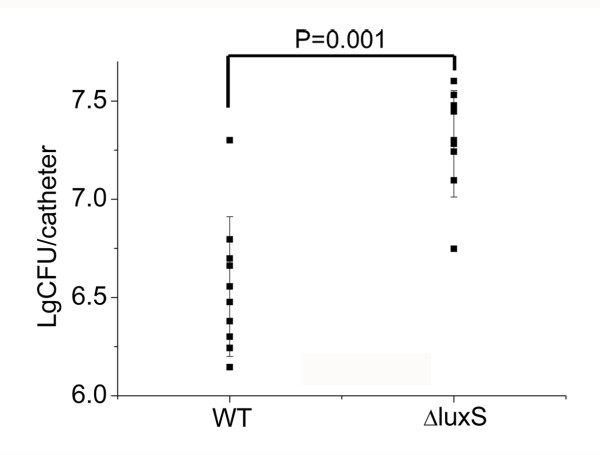
**Biofilm formation of *S. aureus in vivo. *** Biofilm formation was assessed using a murine catheter-associated model of WT (NCTC8325) and ΔluxS (NCTC8325ΔluxS). Overnight culture of 5 × 10^7^ CFU was injected into the catheters, which were implanted subcutaneously in the dorsal area of the mice. Results shown are the number of bacteria counted from the catheters after incubation for 3 days*.* Each point stands for one independent mouse. *P* value refers to a comparison between WT and ΔluxS and means statistically significant differences (P = 0.001) by Student's *t* test.

To further verify the effect of AI-2 on biofilm formation *in vivo*, a murine model of catheter-associated biofilm formation was used. In this assay, mice were separately infected with 5 × 10^7^ CFU/ml of the WT strain and the ΔluxS strain. After incubation for 3 days, the catheters were taken out and the number of bacteria was counted. As shown in Figure 3, the ΔluxS strain exhibited significantly increased capacity to form biofilms compared to the WT strain (P = 0.001) *in vivo*. These results suggest that LuxS/AI-2 system is involved in the regulation of biofilm formation *in vivo*, which is consistent with the conclusion *in vitro*.

### AI-2 represses the transcription of *icaA* via the activation of *icaR*

**Figure 4:**
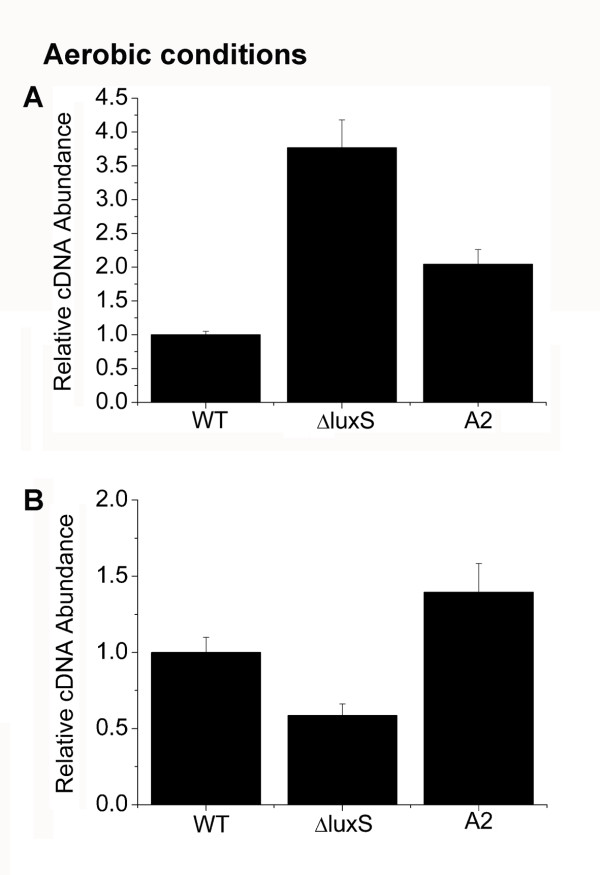
**Transcriptional regulation of *icaA *and *icaR *by AI-2.** Real-time RT-PCR of *icaA* and *icaR* transcription was measured. The bacteria used for RNA extraction were those that were incubated at 4 h for biofilm formation under aerobic conditions. Error bars indicate the variation between triplicate samples within the real-time RT-PCR. The relative cDNA abundance of the WT sample was assigned a value of 1. (**A**) Relative transcript levels of *icaA* of WT (RN6390B), ΔluxS and ΔluxS complemented with 3.9 nM DPD under aerobic conditions. (**B**) Relative transcript levels of *icaR* of WT (RN6390B), ΔluxS and ΔluxS complemented with 3.9 nM DPD under aerobic conditions.

PIA is considered to be a major factor determining biofilm formation in some bacteria [10,54,55]. To test if AI-2-mediated biofilm reduction is due to a change in PIA expression, the transcription of *icaA* was examined using real-time RT-PCR with RNA isolated from biofilm bacteria at different time points. Transcription of *icaA* reached its peak at 4 h of biofilm formation and the maximum difference between the WT strain and the ΔluxS strain was also highlighted at this time (data not shown). Thus, RNA was isolated from 4 h biofilm bacteria of the WT strain, the ΔluxS strain, and the ΔluxS strain complemented with 3.9 nM DPD. Expression of *icaA* was examined using real-time RT-PCR. The resulting data showed that expression of *icaA* was elevated in the ΔluxS strain, and it could be complemented by 3.9 nM DPD (Figure 4A). As expected, corresponding to the biofilm formation in Figure 1, thicker biofilms were presented owing to the *luxS* mutation while the bacteria within the biofilms also displayed elevated *icaA* transcription. Moreover, we examined the expression of several main adhesion molecules. As shown in Additional file 1: Figure S1, there were no obvious differences between the WT, ΔluxS and ΔluxS transformed with the pLIluxS plasmid for complementation (ΔluxSpluxS). Here, the WT and ΔluxS strains were also transformed with an empty PLI50 plasmid constructing the WTp strain and ΔluxSp strain, which were used as the control. Besides, we added sodium-metapeiodate into the well-developed biofilms and found that biofilms dispersed after 2 h incubation at 37°C. Taken together, our results suggest that PIA is the main factor controlled by AI-2 in the regulation of biofilm formation in *S. aureus*.

It was reported that IcaR is a negative regulator of the *icaA* locus [19], and that *icaR* could be regulated by Rbf, SarA and SigB [56,57]. However, few studies indicate that the signalling molecule AI-2 could be an activator of *icaR*. We therefore investigated whether repression of *icaA* by AI-2 was mediated by IcaR by examining the *icaR* transcription in the biofilm bacteria of the WT strain, the ΔluxS strain and the ΔluxS strain complemented with 3.9 nM DPD. We found that the ΔluxS strain displayed decreased transcription of *icaR* compared to WT, and DPD supplementation could complement the effect of *luxS* mutation (Figure 4B). These data indicate that the repression of *icaADBC* transcription by AI-2 is through the activation of *icaR*. These results allow us to conclude that AI-2 activates *icaR*, which results in decreased *icaADBC* transcription and subsequently decreased biofilm formation.

### AI-2 inhibits biofilm formation and represses the transcription of *icaA* under anaerobic conditions

**Figure 5:**
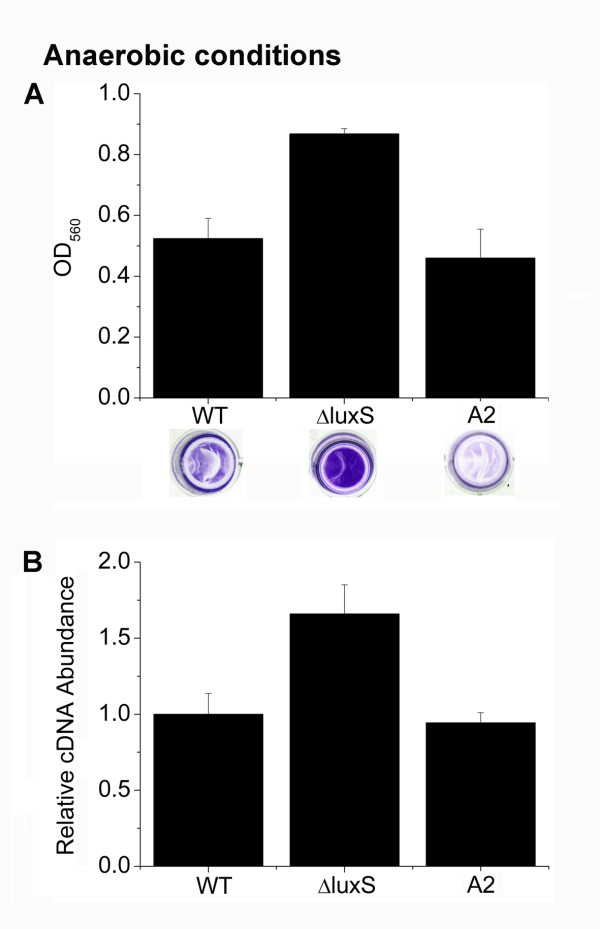
**Analysis of biofilm formation and the *icaA *transcription under anaerobic conditions.** (**A**) Biofilm formation of WT (RN6390B), ΔluxS and ΔluxS complemented with 3.9 nM DPD under anaerobic conditions. (**B**) Relative transcript levels of *icaA* of WT (RN6390B), ΔluxS and ΔluxS complemented with 3.9 nM DPD under anaerobic conditions.

Hypoxia or anaerobic conditions is a common hostile environment that the biofilm bacteria suffer *in vivo*[3,58,59]. To determine whether or not AI-2 could also affect biofilm formation under anaerobic conditions, the microtitre plate assay was used to examine the biofilm growth. After incubation of the plate for 4 h under anaerobic conditions, we found that the ΔluxS strain displayed increased biofilm formation compared to the WT strain, and AI-2 supplementation restored the WT phenotype (Figure 5A). Consistently, AI-2 repressed the transcription of *icaA* under anaerobic conditions (Figure 5B).

### The LuxS/AI-2 QS system and the *agr*-mediated QS system have a cumulative effect on the regulation of biofilm formation

It was reported that the *agr* QS system mediates biofilm dispersal in *S. aureus*[60]. To determine whether the LuxS/AI-2 QS system and the *agr*-mediated QS system have a cumulative or complementary effect on the regulation of biofilm formation, we constructed a Δagr ΔluxS strain and compared the biofilm formation among the WT strain and the mutants using different assays, including the microtitre plate assay, flow cell, anaerobic jar and SEM. Consistently, we found that the Δagr ΔluxS strain displayed the strongest capacity for biofilm formation among all the stains we investigated.

**Figure 6:**
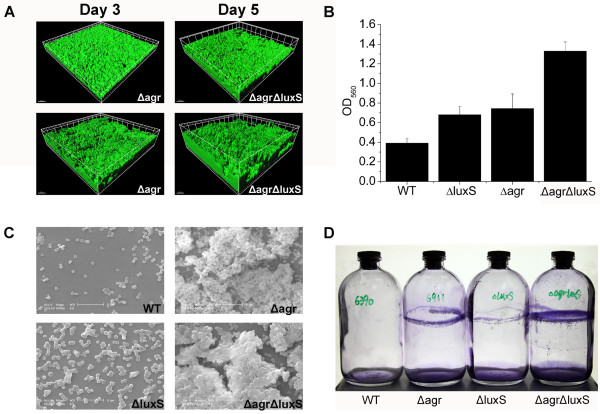
**Additive effect played by the LuxS/AI-2 QS system and the *agr*-mediated QS system.** (**A**) The ΔagrΔluxSG and RN6911G grew biofilms in the flow cell, and the representative images were measured by CLSM at the 3rd and 5th day of biofilm formation. Strains are indicated in the figure. (**B**) Overnight cultures of WT (RN6390B), Δagr (RN6911), ΔluxS and Δagr ΔluxS were inoculated in 24-well plate and formed biofilms under anaerobic conditions. (**C**) WT, Δagr, ΔluxS and Δagr ΔluxS formed 5 days biofilms in a flow cell on the upper surface of the coverslips, which were cut and examined by scanning electron microscopy. (**D**) The anaerobic jar was used for monitoring the biofilm formation of the WT, Δagr, ΔluxS and Δagr ΔluxS, OD_560_ was measured after crystal violet staining.

In the flow-cell assay, as shown in Figure 6A, the Δagr ΔluxS strain formed stronger biofilms than RN6911, as shown by CLSM, indicating that mutation of *luxS* indeed influences biofilm formation and that the two systems seem to play a cumulative effect. Moreover, similar results were obtained in the microtitre plate assay and the anaerobic jar assay under anaerobic conditions (Figure 6B and D).

To accurately describe the distinct biofilm formation resulting from *luxS* deletion, SEM was used for evaluating the structure and surface appearance of the mature biofilm. Therefore, the coverslips of the flow-cell chamber on which 5 days biofilms of WT and the ΔluxS strain grew were cut out. SEM analysis showed that the ΔluxS strain produced a compact biofilm structure with increased coverage than that of the WT strain (Figure 6C). On closer inspection, we found that the ΔluxS strain displayed stronger intercellular adhesion and this was also reflected in the Δagr ΔluxS strain. The Δagr ΔluxS strain showed stronger intercellular adhesion ability than RN6911 (Figure 6C), indicating a possible result of elevated expression of PIA. Interestingly, microscopic analysis of the biofilm structure revealed that the *agr* mutation led to biofilms that adopted a "ridged" appearance with many channels, rather than the relatively smooth, confluent layer normally detected in the WT and ΔluxS strains, presumably because the thicker biofilms with a dense compact structure restrict the growth of bacteria inside. Based on these results, we speculate that the LuxS/AI-2 QS system and the *agr*-mediated QS system play a cumulative effect on the regulation of biofilm formation in *S. aureus*. It has been reported that induction of the *agr* system in established *S. aureus* biofilms detaches cells in an *ica*-independent manner and they also demonstrate that the dispersal mechanism requires extracellular protease activity [60]. Therefore, it seems that the influences of the LuxS/AI-2 QS system and the *agr*-mediated QS system on biofilm formation are through different pathways in *S. aureus*.

## Discussion

Most previous studies of biofilm formation have been performed under one or two conditions to present this phenotype. However, biofilm is a kind of "smart community" that seems able to cope with different environments. Therefore, a single condition may lead to misunderstanding regarding the elaborate function of a specific gene. To provide sufficient and rigorous evidence, we demonstrate that the LuxS/AI-2 system is involved in the regulation of biofilm formation under different conditions. In contrast to the most commonly used microtitre plate assay, flow cell is increasingly used for detecting biofilm growth, of which the dynamic three-dimensional image could be monitored by CLSM dynamically. This setting simulates the environment of flowing surfaces *in vivo*, such as some interfaces between body fluids and implants. The result under this condition may offer more accurate information about flow surroundings *in vivo*. In addition, we also investigated biofilm formation under anaerobic conditions, which the biofilm bacteria undergo. The oxygen partial pressure of air-equilibrated medium is high *in vitro*, whereas hypoxic environments may prevail in body implants and human tissues distant from arterial blood flow [58,61]. Moreover, most locations in which the biofilm bacteria accumulate are usually niches of local low oxygen because of inflammatory cell aggregation [59,62].

The mouse model was used here to compare biofilm formation of the WT and the ΔluxS strains and our data were consistent with the *in vitro* data. Nevertheless, there are few studies regarding AI-2 complementation in the mouse model to date, and the specific mechanism of these signal molecules *in vivo* remains elusive. In general, we offer consistent results under different conditions to emphasise that the conclusion drawn is consistent and worthy of reference in most cases.

LuxS and AI-2 affect biofilm development, whereas the results may be different in the same strain due to various factors. Previous work has shown that AI-2 was produced in rich medium under aerobic and anaerobic conditions peaking during the transition to stationary phase, but cultures retained considerable AI-2 activity after entry into the stationary phase under anaerobic conditions. In addition, the *S. aureus* RN6390BΔluxS strain showed reduction in biofilm formation in TSB containing 1% glucose and 3% sodium chloride under anaerobic conditions [42]. However, in our study, analysis of biofilm growth *in vitro* and *in vivo* led to the conclusion that the ΔluxS strain exhibited increased biofilm formation compared to the WT strain. Importantly, the *luxS* mutation could be complemented by chemically synthesized DPD, indicating the effect of AI-2-mediated QS on biofilm formation in *S. aureus*. Hardie and Heurlier [41] summarised six main factors that influence the experimental results for doing research on the LuxS/AI-2 system: experimental design; genetic complementation; chemical complementation; conditioned supernatant complementation; and complementation with molecules linked to AI-2 production and that independent of *luxS* status. With detailed analysis, we found that the inconsistency of the results is in part owing to the different growth medium provided to the biofilm bacteria, especially the different concentrations of glucose and sodium chloride, which are both important factors enhancing biofilm formation [63].

In addition to the present evidence of AI-2-regulated biofilm formation in *S. aureus*, we found that the transcription of *icaR* is activated by AI-2, which is barely reported, although we have not yet identified the mechanism of the interaction between them. This is partly because the detailed mechanism of transport and action of AI-2 has only been described in several strains and different mechanisms are applied to different species, although AI-2 has been proven to act as a signalling molecule that could regulate series of gene expression. The first mechanism revealed was in *Vibrio harveyi*, which responds to AI-2 by initiating a phosphorylation cascade [64]. In *Salmonella typhimurium*[65] and *E. coli*[66,67], AI-2 seems to be taken up by an ABC transporter. However, the mechanism of AI-2 transport and functional performing in *Staphylococci* was still unknown*.* Therefore, the detailed mechanism through which AI-2 functions in *S. aureus* should be highlighted here, and the interaction between AI-2 and IcaR requires further study.

In addition to PIA, we do not observe any obvious increase of extracellular protein (Additional file 2: Figure S2) or bacterial autolysis in the ΔluxS strain (Additional file 3: Figure S3). Our results showed no significant differences in the transcriptional levels of several main adhesion molecules. Moreover, previous work indicated that *S. aureus* strains 8325-4 and RN4220 formed PIA-dependent biofilms [68]. We thus propose that AI-2 signalling represses the *icaA* expression, and subsequently leads to decreased biofilm formation in *S. aureus*.

More and more studies concerning multispecies biofilms gradually uncover the mechanisms of the interaction and communication of the different species inside the biofilms. One of the most popular approaches of the signalling regulation is directed towards the AI-2-controlled QS system for its extensive use of interspecies. The plaque biofilms on tooth surfaces consist of various oral bacteria including *S. aureus* and involve complex microbial interactions [69-71]. There is evidence that AI-2-mediated QS may play a significant role in oral biofilm formation [50]. As reported by others, airway infections by *Pseudomonas aeruginosa* afflicting patients with cystic fibrosis (CF) are among the most enigmatic of biofilm diseases [2]. *S. aureus* is also found to be a major pathogen associated with *P. aeruginosa* in CF lung infection [72]. Previous work has shown that PIA is expressed in lungs infected with *S. aureus*, whereas CP8 is not expressed because of limited oxygen [73]. Here, we provide evidence that AI-2 can regulate *icaA* expression under anaerobic conditions, suggesting a potential role of AI-2 in influencing *S. aureus* infection in lungs. However, few studies about biofilm formation cooperated by *S. aureus* and the other species are reported. Therefore, could *S. aureus* and the other species in their focus areas form multispecies biofilms? Could AI-2 play an important role in this process? It is interesting to discuss the actual complex-flora interaction in human and social behaviour of the bacteria. Therefore, revelation of the AI-2-regulated biofilm formation in *S. aureus* possesses instructive meaning for these related studies.

## Conclusions

These findings demonstrate that AI-2 can decrease biofilm formation in *S. aureus* via an *icaR*-activation pathway. This study may provide clues for therapy in *S. aureus* biofilm-associated infection.

## Competing interests

The authors declare that they have no competing interests.

## Authors’ contribution

DY carried out the experiments and performed the data analyses. BS, ZL, and TX contributed to the design and coordination of the experiments. DY wrote the manuscript. BS, TX and ZL participated in editing the manuscript. All authors have read and approved the manuscript.

## Supplementary Material

Additional file 1 **Relative transcript levels of several adhesions.** The levels of transcription of these genes including *map*, *fnbA*, *fnbB*, *clfB*, *efb* were measured by real-time RT-PCR in *S. aureus* WTp, ΔluxSp and ΔluxS complemented with a plasmid containing *luxS* gene for genetic complementation (ΔluxSpluxS). As the control, WT and ΔluxS were transformed with empty plasmid PLI50, constructing WTp and ΔluxSp. Click here for file

Additional file 2 **Extracellular protein loaded on SDS-PAGE.** The levels of extracellular-protein expression of biofilm bacteria, which were incubated at 37°C for 4 h and 24 h, were measured. Click here for file

Additional file 3 **Triton X-100-stimulated autolysis.** The autolysis of WT, ΔluxS and ΔluxSpluxS induced in 0.05 M Tris–HCl buffer containing 0.05% (vol/vol) Triton X-100 were measured. Click here for file

## References

[B1] Harris LG Richards RG Staphylococci and implant surfaces: a review Injury 2006 37 Suppl 2 S3 S14 16651069 10.1016/j.injury.2006.04.003

[B2] Parsek MR Singh PK Bacterial biofilms: an emerging link to disease pathogenesis Annu Rev Microbiol 2003 57 677 701 10.1146/annurev.micro.57.030502.090720 14527295

[B3] Cooper R Okhiria O Biofilms, wound infection and the issue of control Wounds UK 2006 2 3 48 56

[B4] Costerton JW Stewart PS Greenberg EP Bacterial biofilms: a common cause of persistent infections Science 1999 284 5418 1318 1322 10.1126/science.284.5418.1318 10334980

[B5] Otto M Staphylococcal biofilms Curr Top Microbiol Immunol 2008 322 207 228 10.1007/978-3-540-75418-3_10 18453278 PMC2777538

[B6] Rice KC Mann EE Endres JL Weiss EC Cassat JE Smeltzer MS Bayles KW The cidA murein hydrolase regulator contributes to DNA release and biofilm development in Staphylococcus aureus Proc Natl Acad Sci USA 2007 104 19 8113 8118 10.1073/pnas.0610226104 17452642 PMC1876580

[B7] Izano EA Amarante MA Kher WB Kaplan JB Differential roles of poly-N-acetylglucosamine surface polysaccharide and extracellular DNA in Staphylococcus aureus and Staphylococcus epidermidis biofilms Appl Environ Microbiol 2008 74 2 470 476 10.1128/AEM.02073-07 18039822 PMC2223269

[B8] Heilmann C Gerke C Perdreau-Remington F Gotz F Characterization of Tn917 insertion mutants of Staphylococcus epidermidis affected in biofilm formation Infect Immun 1996 64 1 277 282 8557351 10.1128/iai.64.1.277-282.1996 PMC173756

[B9] Heilmann C Gotz F Further characterization of Staphylococcus epidermidis transposon mutants deficient in primary attachment or intercellular adhesion Zentralbl Bakteriol 1998 287 1–2 69 83 9532266 10.1016/s0934-8840(98)80149-7

[B10] Mack D Fischer W Krokotsch A Leopold K Hartmann R Egge H Laufs R The intercellular adhesin involved in biofilm accumulation of Staphylococcus epidermidis is a linear beta-1,6-linked glucosaminoglycan: purification and structural analysis J Bacteriol 1996 178 1 175 183 8550413 10.1128/jb.178.1.175-183.1996 PMC177636

[B11] Heilmann C Schweitzer O Gerke C Vanittanakom N Mack D Gotz F Molecular basis of intercellular adhesion in the biofilm-forming Staphylococcus epidermidis Mol Microbiol 1996 20 5 1083 1091 10.1111/j.1365-2958.1996.tb02548.x 8809760

[B12] Gerke C Kraft A Sussmuth R Schweitzer O Gotz F Characterization of the N-acetylglucosaminyltransferase activity involved in the biosynthesis of the Staphylococcus epidermidis polysaccharide intercellular adhesin J Biol Chem 1998 273 29 18586 18593 10.1074/jbc.273.29.18586 9660830

[B13] Cramton SE Gerke C Schnell NF Nichols WW Gotz F The intercellular adhesion (ica) locus is present in Staphylococcus aureus and is required for biofilm formation Infect Immun 1999 67 10 5427 5433 10496925 10.1128/iai.67.10.5427-5433.1999 PMC96900

[B14] Mack D Siemssen N Laufs R Parallel induction by glucose of adherence and a polysaccharide antigen specific for plastic-adherent Staphylococcus epidermidis: evidence for functional relation to intercellular adhesion Infect Immun 1992 60 5 2048 2057 1314224 10.1128/iai.60.5.2048-2057.1992 PMC257114

[B15] Campbell IM Crozier DN Pawagi AB Buivids IA In vitro response of Staphylococcus aureus from cystic fibrosis patients to combinations of linoleic and oleic acids added to nutrient medium J Clin Microbiol 1983 18 2 408 415 6619290 10.1128/jcm.18.2.408-415.1983 PMC270814

[B16] Hjelm E Lundell-Etherden I Slime production by Staphylococcus saprophyticus Infect Immun 1991 59 1 445 448 1987058 10.1128/iai.59.1.445-448.1991 PMC257762

[B17] Cramton SE Ulrich M Gotz F Doring G Anaerobic conditions induce expression of polysaccharide intercellular adhesin in Staphylococcus aureus and Staphylococcus epidermidis Infect Immun 2001 69 6 4079 4085 10.1128/IAI.69.6.4079-4085.2001 11349079 PMC98472

[B18] Deighton M Borland R Regulation of slime production in Staphylococcus epidermidis by iron limitation Infect Immun 1993 61 10 4473 4479 8406839 10.1128/iai.61.10.4473-4479.1993 PMC281182

[B19] Jefferson KK Pier DB Goldmann DA Pier GB The teicoplanin-associated locus regulator (TcaR) and the intercellular adhesin locus regulator (IcaR) are transcriptional inhibitors of the ica locus in Staphylococcus aureus J Bacteriol 2004 186 8 2449 2456 10.1128/JB.186.8.2449-2456.2004 15060048 PMC412131

[B20] Xu L Li H Vuong C Vadyvaloo V Wang J Yao Y Otto M Gao Q Role of the luxS quorum-sensing system in biofilm formation and virulence of Staphylococcus epidermidis Infect Immun 2006 74 1 488 496 10.1128/IAI.74.1.488-496.2006 16369005 PMC1346618

[B21] Bassler BL How bacteria talk to each other: regulation of gene expression by quorum sensing Curr Opin Microbiol 1999 2 6 582 587 10.1016/S1369-5274(99)00025-9 10607620

[B22] Miller MB Bassler BL Quorum sensing in bacteria Annu Rev Microbiol 2001 55 165 199 10.1146/annurev.micro.55.1.165 11544353

[B23] De Keersmaecker SC Sonck K Vanderleyden J Let LuxS speak up in AI-2 signaling Trends Microbiol 2006 14 3 114 119 10.1016/j.tim.2006.01.003 16459080

[B24] Surette MG Miller MB Bassler BL Quorum sensing in Escherichia coli, Salmonella typhimurium, and Vibrio harveyi: a new family of genes responsible for autoinducer production Proc Natl Acad Sci USA 1999 96 4 1639 1644 10.1073/pnas.96.4.1639 9990077 PMC15544

[B25] Schauder S Shokat K Surette MG Bassler BL The LuxS family of bacterial autoinducers: biosynthesis of a novel quorum-sensing signal molecule Mol Microbiol 2001 41 2 463 476 10.1046/j.1365-2958.2001.02532.x 11489131

[B26] Chen X Schauder S Potier N Van Dorsselaer A Pelczer I Bassler BL Hughson FM Structural identification of a bacterial quorum-sensing signal containing boron Nature 2002 415 6871 545 549 10.1038/415545a 11823863

[B27] Miller ST Xavier KB Campagna SR Taga ME Semmelhack MF Bassler BL Hughson FM Salmonella typhimurium recognizes a chemically distinct form of the bacterial quorum-sensing signal AI-2 Mol Cell 2004 15 5 677 687 10.1016/j.molcel.2004.07.020 15350213

[B28] Lupp C Ruby EG Vibrio fischeri LuxS and AinS: comparative study of two signal synthases J Bacteriol 2004 186 12 3873 3881 10.1128/JB.186.12.3873-3881.2004 15175301 PMC419941

[B29] Rader BA Campagna SR Semmelhack MF Bassler BL Guillemin K The quorum-sensing molecule autoinducer 2 regulates motility and flagellar morphogenesis in Helicobacter pylori J Bacteriol 2007 189 17 6109 6117 10.1128/JB.00246-07 17586631 PMC1951907

[B30] Bansal T Jesudhasan P Pillai S Wood TK Jayaraman A Temporal regulation of enterohemorrhagic Escherichia coli virulence mediated by autoinducer-2 Appl Microbiol Biotechnol 2008 78 5 811 819 10.1007/s00253-008-1359-8 18256823

[B31] Gonzalez Barrios AF Zuo R Hashimoto Y Yang L Bentley WE Wood TK Autoinducer 2 controls biofilm formation in Escherichia coli through a novel motility quorum-sensing regulator (MqsR, B3022) J Bacteriol 2006 188 1 305 316 10.1128/JB.188.1.305-316.2006 16352847 PMC1317603

[B32] Shao H Lamont RJ Demuth DR Autoinducer 2 is required for biofilm growth of Aggregatibacter (Actinobacillus) actinomycetemcomitans Infect Immun 2007 75 9 4211 4218 10.1128/IAI.00402-07 17591788 PMC1951166

[B33] Vidal JE Ludewick HP Kunkel RM Zahner D Klugman KP The LuxS-dependent quorum-sensing system regulates early biofilm formation by Streptococcus pneumoniae strain D39 Infect Immun 2011 79 10 4050 4060 10.1128/IAI.05186-11 21825061 PMC3187250

[B34] Auger S Krin E Aymerich S Gohar M Autoinducer 2 affects biofilm formation by Bacillus cereus Appl Environ Microbiol 2006 72 1 937 941 10.1128/AEM.72.1.937-941.2006 16391139 PMC1352198

[B35] Tannock GW Ghazally S Walter J Loach D Brooks H Cook G Surette M Simmers C Bremer P Dal Bello F Ecological behavior of Lactobacillus reuteri 100–23 is affected by mutation of the luxS gene Appl Environ Microbiol 2005 71 12 8419 8425 10.1128/AEM.71.12.8419-8425.2005 16332830 PMC1317450

[B36] Challan Belval S Gal L Margiewes S Garmyn D Piveteau P Guzzo J Assessment of the roles of LuxS, S-ribosyl homocysteine, and autoinducer 2 in cell attachment during biofilm formation by Listeria monocytogenes EGD-e Appl Environ Microbiol 2006 72 4 2644 2650 10.1128/AEM.72.4.2644-2650.2006 16597969 PMC1449078

[B37] Lebeer S De Keersmaecker SC Verhoeven TL Fadda AA Marchal K Vanderleyden J Functional analysis of luxS in the probiotic strain Lactobacillus rhamnosus GG reveals a central metabolic role important for growth and biofilm formation J Bacteriol 2007 189 3 860 871 10.1128/JB.01394-06 17098890 PMC1797292

[B38] Learman DR Yi H Brown SD Martin SL Geesey GG Stevens AM Hochella MF Jr Involvement of Shewanella oneidensis MR-1 LuxS in biofilm development and sulfur metabolism Appl Environ Microbiol 2009 75 5 1301 1307 10.1128/AEM.01393-08 19124589 PMC2648156

[B39] De Keersmaecker SCJ Varszegi C van Boxel N Habel LW Metzger K Daniels R Marchal K De Vos D Vanderleyden J Chemical synthesis of (S)-4,5-dihydroxy-2,3-pentanedione, a bacterial signal molecule precursor, and validation of its activity in Salmonella typhimurium J Biol Chem 2005 280 20 19563 19568 10.1074/jbc.M412660200 15790567

[B40] Li L Xu Z Zhou Y Li T Sun L Chen H Zhou R Analysis on Actinobacillus pleuropneumoniae LuxS regulated genes reveals pleiotropic roles of LuxS/AI-2 on biofilm formation, adhesion ability and iron metabolism Microb Pathog 2011 50 6 293 302 10.1016/j.micpath.2011.02.002 21320583

[B41] Hardie KR Heurlier K Establishing bacterial communities by 'word of mouth': LuxS and autoinducer 2 in biofilm development Nat Rev Microbiol 2008 6 8 635 643 10.1038/nrmicro1916 18536728

[B42] Doherty N Holden MT Qazi SN Williams P Winzer K Functional analysis of luxS in Staphylococcus aureus reveals a role in metabolism but not quorum sensing J Bacteriol 2006 188 8 2885 2897 10.1128/JB.188.8.2885-2897.2006 16585750 PMC1446992

[B43] Zhao L Xue T Shang F Sun H Sun B Staphylococcus aureus AI-2 quorum sensing associates with the KdpDE two-component system to regulate capsular polysaccharide synthesis and virulence Infect Immun 2010 78 8 3506 3515 10.1128/IAI.00131-10 20498265 PMC2916273

[B44] Kuehl R Al-Bataineh S Gordon O Luginbuehl R Otto M Textor M Landmann R Furanone at subinhibitory concentrations enhances staphylococcal biofilm formation by luxS repression Antimicrob Agents Chemother 2009 53 10 4159 4166 10.1128/AAC.01704-08 19620329 PMC2764226

[B45] Bruckner R Gene replacement in Staphylococcus carnosus and Staphylococcus xylosus FEMS Microbiol Lett 1997 151 1 1 8 10.1016/S0378-1097(97)00116-X 9198277

[B46] Beenken KE Blevins JS Smeltzer MS Mutation of sarA in Staphylococcus aureus limits biofilm formation Infect Immun 2003 71 7 4206 4211 10.1128/IAI.71.7.4206-4211.2003 12819120 PMC161964

[B47] Yarwood JM Bartels DJ Volper EM Greenberg EP Quorum sensing in Staphylococcus aureus biofilms J Bacteriol 2004 186 6 1838 1850 10.1128/JB.186.6.1838-1850.2004 14996815 PMC355980

[B48] Heydorn A Nielsen AT Hentzer M Sternberg C Givskov M Ersboll BK Molin S Quantification of biofilm structures by the novel computer program COMSTAT Microbiology 2000 146 Pt 10 2395 2407 11021916 10.1099/00221287-146-10-2395

[B49] Beenken KE Dunman PM McAleese F Macapagal D Murphy E Projan SJ Blevins JS Smeltzer MS Global gene expression in Staphylococcus aureus biofilms J Bacteriol 2004 186 14 4665 4684 10.1128/JB.186.14.4665-4684.2004 15231800 PMC438561

[B50] Yoshida A Ansai T Takehara T Kuramitsu HK LuxS-based signaling affects Streptococcus mutans biofilm formation Appl Environ Microbiol 2005 71 5 2372 2380 10.1128/AEM.71.5.2372-2380.2005 15870324 PMC1087550

[B51] Rickard AH Palmer RJ Blehert DS Campagna SR Semmelhack MF Egland PG Bassler BL Kolenbrander PE Autoinducer 2: a concentration-dependent signal for mutualistic bacterial biofilm growth Mol Microbiol 2006 60 6 1446 1456 10.1111/j.1365-2958.2006.05202.x 16796680

[B52] Feather MS Amine-assisted sugar dehydration reactions Prog Food Nutr Sci 1981 5 37 45

[B53] Nedvidek W Ledl F Fischer P Detection of 5-hydroxymethyl-1-2-methyl-3(2H)-furanone and of α-dicarbonyl compounds in reaction mixtures of hexoses and pentoses with different amines Z Lebensm UntersForsch 1992 194 222 228 10.1007/BF01198411

[B54] Gotz F Staphylococcus and biofilms Mol Microbiol 2002 43 6 1367 1378 10.1046/j.1365-2958.2002.02827.x 11952892

[B55] Mack D Haeder M Siemssen N Laufs R Association of biofilm production of coagulase-negative staphylococci with expression of a specific polysaccharide intercellular adhesin J Infect Dis 1996 174 4 881 884 10.1093/infdis/174.4.881 8843236

[B56] Cue D Lei MG Luong TT Kuechenmeister L Dunman PM O'Donnell S Rowe S O'Gara JP Lee CY Rbf promotes biofilm formation by Staphylococcus aureus via repression of icaR, a negative regulator of icaADBC J Bacteriol 2009 191 20 6363 6373 10.1128/JB.00913-09 19684134 PMC2753044

[B57] Cerca N Brooks JL Jefferson KK Regulation of the intercellular adhesin locus regulator (icaR) by SarA, sigmaB, and IcaR in Staphylococcus aureus J Bacteriol 2008 190 19 6530 6533 10.1128/JB.00482-08 18658265 PMC2565999

[B58] Coleman G Garbutt IT Demnitz U Ability of a Staphylococcus aureus isolate from a chronic osteomyelitic lesion to survive in the absence of air Eur J Clin Microbiol 1983 2 6 595 597 10.1007/BF02016574 6667685

[B59] Simmen HP Blaser J Analysis of pH and pO2 in abscesses, peritoneal fluid, and drainage fluid in the presence or absence of bacterial infection during and after abdominal surgery Am J Surg 1993 166 1 24 27 10.1016/S0002-9610(05)80576-8 8328625

[B60] Boles BR Horswill AR Agr-mediated dispersal of Staphylococcus aureus biofilms PLoS Pathog 2008 4 4 e1000052 10.1371/journal.ppat.1000052 18437240 PMC2329812

[B61] Ernst JF Tielker D Responses to hypoxia in fungal pathogens Cell Microbiol 2009 11 2 183 190 10.1111/j.1462-5822.2008.01259.x 19016786

[B62] McGovern NN Cowburn AS Porter L Walmsley SR Summers C Thompson AA Anwar S Willcocks LC Whyte MK Condliffe AM Hypoxia selectively inhibits respiratory burst activity and killing of Staphylococcus aureus in human neutrophils J Immunol 2011 186 1 453 463 10.4049/jimmunol.1002213 21135168 PMC4374781

[B63] Moretro T Hermansen L Holck AL Sidhu MS Rudi K Langsrud S Biofilm formation and the presence of the intercellular adhesion locus ica among staphylococci from food and food processing environments Appl Environ Microbiol 2003 69 9 5648 5655 10.1128/AEM.69.9.5648-5655.2003 12957956 PMC194930

[B64] Bassler BL Wright M Silverman MR Sequence and function of LuxO, a negative regulator of luminescence in Vibrio harveyi Mol Microbiol 1994 12 3 403 412 10.1111/j.1365-2958.1994.tb01029.x 8065259

[B65] Taga ME Miller ST Bassler BL Lsr-mediated transport and processing of AI-2 in Salmonella typhimurium Mol Microbiol 2003 50 4 1411 1427 10.1046/j.1365-2958.2003.03781.x 14622426

[B66] Wang L Hashimoto Y Tsao CY Valdes JJ Bentley WE Cyclic AMP (cAMP) and cAMP receptor protein influence both synthesis and uptake of extracellular autoinducer 2 in Escherichia coli J Bacteriol 2005 187 6 2066 2076 10.1128/JB.187.6.2066-2076.2005 15743955 PMC1064054

[B67] Xavier KB Bassler BL Regulation of uptake and processing of the quorum-sensing autoinducer AI-2 in Escherichia coli J Bacteriol 2005 187 1 238 248 10.1128/JB.187.1.238-248.2005 15601708 PMC538819

[B68] O'Neill E Pozzi C Houston P Smyth D Humphreys H Robinson DA O'Gara JP Association between methicillin susceptibility and biofilm regulation in Staphylococcus aureus isolates from device-related infections J Clin Microbiol 2007 45 5 1379 1388 10.1128/JCM.02280-06 17329452 PMC1865887

[B69] Kolenbrander PE Andersen RN Blehert DS Egland PG Foster JS Palmer RJ Jr Communication among oral bacteria Microbiol Mol Biol Rev 2002 66 3 486 505 table of contents 10.1128/MMBR.66.3.486-505.2002 12209001 PMC120797

[B70] Didilescu AC Skaug N Marica C Didilescu C Respiratory pathogens in dental plaque of hospitalized patients with chronic lung diseases Clin Oral Investig 2005 9 3 141 147 10.1007/s00784-005-0315-6 15909174

[B71] Sumi Y Miura H Michiwaki Y Nagaosa S Nagaya M Colonization of dental plaque by respiratory pathogens in dependent elderly Arch Gerontol Geriatr 2007 44 2 119 124 10.1016/j.archger.2006.04.004 16723159

[B72] Govan JR Infection control in cystic fibrosis: methicillin-resistant Staphylococcus aureus, Pseudomonas aeruginosa and the Burkholderia cepacia complex J R Soc Med 2000 93 Suppl 38 40 45 10911818 PMC1305883

[B73] McKenney D Pouliot KL Wang Y Murthy V Ulrich M Doring G Lee JC Goldmann DA Pier GB Broadly protective vaccine for Staphylococcus aureus based on an in vivo-expressed antigen Science 1999 284 5419 1523 1527 10.1126/science.284.5419.1523 10348739

