# Supplementary figures and images for: Staphylococcus aureus autoinducer-2 quorum sensing decreases biofilm formation in an icaR-dependent manner
Source: BMC Microbiol. 2012 Dec 5;12:288. doi: 10.1186/1471-2180-12-288 (PMC3539994; doi:10.1186/1471-2180-12-288)

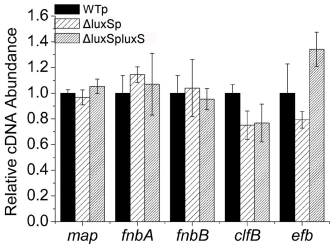

Supplement: Additional file 1 — Relative transcript levels of several adhesions. The levels of transcription of these genes including map, fnbA, fnbB, clfB, efb were measured by real-time RT-PCR in S. aureus WTp, ΔluxSp and ΔluxS complemented with a plasmid containing luxS gene for genetic complementation (ΔluxSpluxS). As the control, WT and ΔluxS were transformed with empty plasmid PLI50, constructing WTp and ΔluxSp. [file 1471-2180-12-288-S1.pdf]

WTp  $\Delta$ luxSp  $\Delta$ luxSpluxS

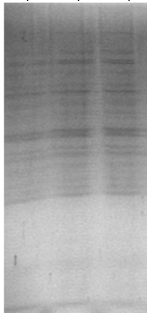

4h

WTp  $\Delta$ luxSp  $\Delta$ luxSpluxS

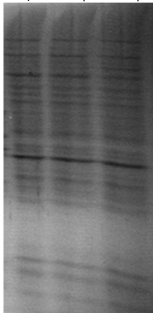

24h

Supplement: Additional file 2 — Extracellular protein loaded on SDS-PAGE. The levels of extracellular-protein expression of biofilm bacteria, which were incubated at 37°C for 4 h and 24 h, were measured. [file 1471-2180-12-288-S2.pdf]

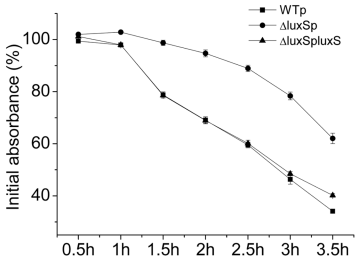

Supplement: Additional file 3 — Triton X-100-stimulated autolysis. The autolysis of WT, ΔluxS and ΔluxSpluxS induced in 0.05 M Tris–HCl buffer containing 0.05% (vol/vol) Triton X-100 were measured. [file 1471-2180-12-288-S3.pdf]
